# Lifestyle factors associated with poor sleep quality among undergraduate dental students at a Malaysian private university

**DOI:** 10.5935/1984-0063.20220070

**Published:** 2022

**Authors:** Hasnah Hashim, Jiong Sen Ng, Jin Xuan Ngo, Yong Zhi Ng, Balaraman Aravindkumar

**Affiliations:** 1 AIMST University, Faculty of Dentistry - Bedong - Kedah - Malaysia; 2 Parit Baru Dental Clinic, Sabak Bernam - Selangor - Malaysia; 3 Presinct 18 Dental Clinic, Putrajaya - Federal Territory of Putrajaya - Malaysia; 4 Pasir Pinji Dental Clinic, Ipoh, Kinta - Perak - Malaysia

**Keywords:** Students, Dental, Sleep, Lifestyle, Malaysia

## Abstract

**Objective:**

The purpose of this study was to look into the associations between lifestyle factors, gender, clinical level, and sleep quality among undergraduate dental students at a private university in Malaysia.

**Material and Methods:**

A self-administered Pittsburg sleep quality index (PSQI) scale and the lifestyle and habits questionnaire-brief (LHQ-B) were used in this cross-sectional study. A global PSQI score of greater than 5 indicates poor sleep quality. All university dental students were invited to take part. Descriptive statistics and logistic regression analyses were used to analyze the data.

**Results:**

A total of 338 students took part in the study, with a response rate of 90.4%. The proportion of females was higher (68.3 %) and more than half of the respondents (56.7 %) were in their clinical years. The prevalence of poor sleep quality was 36.7%. At multivariable level, poor sleep quality was associated with being male (OR=1.72 [95% confidence interval (1.05, 2.83)] and engaging in an unhealthy lifestyle for psychological health (OR=2.64 [95% confidence interval (1.34, 5.21)] and nutrition (OR=2.48 [95% confidence interval (1.028, 4.82)].

**Conclusion:**

The prevalence of poor sleep quality among undergraduate dental students in our study was comparable to that found in other studies. Male students were more likely to have poor sleep quality than female students. Our findings indicate that poor sleep quality (PSQI score >5) may be linked to unhealthy lifestyle habits related to psychological health and nutrition. Health education that emphasizes these domains is essential for improving their lifestyle habits and sleep quality.

## INTRODUCTION

Sleep is an essential component of life. Various theories on the functions of sleep have been proposed, including the notion that it is a period of total body and neurological restoration or recuperation^1^. As a result, a good night’s sleep ensures that a person wakes up feeling refreshed and better prepared to face the day’s activities.

A growing body of evidence suggests that sleep problems are common among university students. A study of over 2,800 students enrolled in 19 universities across Luxembourg and Germany discovered that 42.8% of respondents reported experiencing poor sleep quality in the preceding month^2^. Research focusing on health professional courses found a similar problem among the students. Elagra et al. (2016)^3^, for example, investigated sleep quality among dental undergraduates at a Saudi Arabian university and discovered a 72.5% prevalence. Likewise, a study of medical students in Egypt found that 58.5% of their subjects had poor sleep quality^4^. This is a cause for concern because insufficient sleep quality has been linked to poor academic performance^5-7^.

Sleep deprivation has been associated with lifestyle factors in youths, particularly university students^8,9^. While regular aerobic exercise can improve sleep quality^10^, the mechanisms by which physical exercise affects sleep remain controversial. Theoretical explanations for the link between physical exercise and sleep quality include thermoregulatory adaptation^11^ and an increase in interleukin-1 (IL-1), interleukin-6 (IL-6), and tumor necrosis factor (TNF) after exercise, which may alter sleep^12^.

Psychological distress has a negative impact on sleep quality in college students. Alotaibi et al. (2020)^13^ examined the relationship between stress and sleep quality in Saudi preclinical medical students and discovered a significant inverse relationship between the two variables. This finding supports a Pakistani study that found dental students with high-stress levels struggled to get a good night’s sleep^14^. Medical and dental education are often recognized as being extremely tough for students. In addition to the rigorous training, dental undergraduates are expected to learn specific psychomotor skills required in dentistry. Gender, an intense academic curriculum, the pressure to complete a certain number of clinical cases in a limited amount of time, examinations and grades, fear of academic failure, and uncertain job employment have all been linked to stress among dental students^15-17^. Babar et al. (2015)^18^ confirmed these factors when they investigated perceived stressors among Malaysian dental students. Moreover, they discovered that dental students attending private universities appeared to be more concerned about unemployment than those at public universities, possibly due to the need to repay higher student loans after graduation.

Transitioning from high school to college life frequently involves significant dietary changes. Students’ hectic daily schedules can make it difficult for them to eat nutritious meals regularly, and as a result, many end up consuming more fast food and snacks despite knowing the negative effects of eating a poor-quality diet^19^. In agreement, a previous study of non-nutrition major university students in Saudi Arabia reported that the main barriers to eating fruits and vegetables were a lack of time to prepare meals and a lack of access to nutritious foods on campus^20^. It has also been discovered that eating nutritious foods improves sleep quality while eating processed foods impairs it^21^. To substantiate this, Nisar et al. (2019)^22^ investigated medical students’ dietary intake and its impact on their sleep quality, and they established a link between poor diet and reduced sleep quality.

Several studies have been conducted to ascertain the relationship between alcohol, illicit drugs, and sleep. According to a study of college students in the United States, alcohol use/cigarette smoking significantly predicted poor sleep, but there the authors did not discover a significant link between illegal drug use, such as opiates and methamphetamines, and sleep quality^23^. A study in the Middle East similarly discovered a strong association between substance use and sleep quality among Saudi Arabian and Yemeni university students, however, cross-sectional data cannot be utilized to infer causal conclusions^24^.

Sleep quality is influenced by the quality of one’s social relationships. According to Jin et al. (2014)^25^, having good social support from friends, family, and classmates has a positive effect on sleep status among Chinese undergraduate students. It was hypothesized that a strong support system provides a greater sense of meaning in life, a clear sense of purpose, and positive personal functionality, all of which contribute to improved sleep^26^.

As a result, the purpose of this study is to determine if the following lifestyle domains: physical health and exercise, psychological health, substance use, nutrition, social concern, and sense of purpose, have an effect on the sleep quality of undergraduate dental students at a private institution in Malaysia. We also looked into whether the gender and clinical phase of the students were related to the outcome variable.

## MATERIAL AND METHODS

This cross-sectional study employed two sets of self-administered English questionnaires, the Pittsburg sleep quality index (PSQI) and the lifestyle and habits questionnaire-brief (LHQ-B). The developers of the instruments granted permission.

### Sleep quality

PSQI is a self-administered 19-item scale used in epidemiological and clinical studies around the world to assess subjective sleep quality over the previous month^27^. This questionnaire contains 19 items with seven component scores: (1) sleep quality; (2) sleep latency; (3) sleep duration; (4) sleep efficiency; (5) sleep disturbance; (6) medication use; and (7) daytime dysfunction. The sum of these seven scores is known as the global score, and higher scores indicate poorer sleep quality. PSQI was used in this study because it has been shown to have good discriminant validity between poor and good sleep, with a sensitivity of 89.6% and a specificity of 86.5% when the global score cutoff was greater than 5. The internal consistency of the scale in our sample, as measured by Cronbach’s alpha, was 0.56, indicating moderate consistency^28^.

### Lifestyle

LHQ-B items were used to determine if lifestyle factors are related to sleep quality. The LHQ-B is a 42-item questionnaire that assesses eight lifestyle domains: health and exercise, psychological health, substance use, nutrition, environmental concern, social concern, accident prevention/safety, and sense of purpose^29^. Among college students aged 18 to 25 years, each domain has been shown to have significant to nearly perfect internal consistency reliability (Cronbach’s alpha 0.65 to 0.91). The scale employs a five-point Likert scale, ranging from 1 (strongly disagree) to 5 (strongly agree). Higher totaled scores in the respective domains indicate a healthier lifestyle, whereas lower totaled scores indicate a negative lifestyle. We excluded environmental concerns and accident prevention/safety components in this study because these domain items were considered to be less suitable for the study population, and thus only the remaining six areas of LHQ-B were used to assess lifestyle.

### Data collection

This cross-sectional study invited all 374 undergraduate dental students enrolled at the university to participate voluntarily. Students were given a study information sheet, a consent form, and the PSQI and LHQ-B questionnaires to complete, and those who agreed to participate signed the consent forms. The university’s Human and Animal Ethics Committee approved the study protocol (ref. AUHEC/FOD/2019/18).

### Data analysis

Sleep quality was classified as “good” (PSQI scores £5) or “poor” (PSQI scores >5). Clinical levels were classified as “preclinical” (years 1 and 2) and “clinical” (year 3, year 4, and year 5). Lifestyle behaviors were classified as “healthy”, “moderate” or “unhealthy” based on the developers’ interpretive score cutoff guidelines for male and female subjects (Table 1).

**Table 1:** Interpretive guidelines for the lifestyle and habits questionnaire-brief (LHQ-B).

	Unhealthy		Moderate		Healthy	
	**Male**	**Female**	**Male**	**Female**	**Male**	**Female**
Health and exercise	≤ 19	≤ 15	20-24	16-21	≥ 25	≥ 22
Psychological health	≤ 24	≤ 23	25-29	24-27	≥ 30	≥ 28
Substance use	≤ 25	≤ 29	26-33	30-35	≥ 34	≥ 36
Nutrition	≤ 9	≤ 10	10-12	11-13	≥ 13	≥ 14
Social concern	≤ 19	≤ 19	20-22	20-22	≥ 23	≥ 23
Sense of purpose	≤ 10	≤ 11	11-13	12-13	≥ 14	≥ 14

Categorical variables were summarized using frequency and percentages, with 95% exact binomial confidence intervals (95 percent CI) for the proportions. The relationships between the independent variables and the outcome variable (sleep quality) were explored using univariable and multiple logistic regressions, which yielded crude and adjusted odds ratios, respectively. All independent variables, regardless of their *p*-values in univariable logistic regression analysis, were included in the multivariable selection procedure. The preliminary main-effect model was generated using automatic variable selection methods with stepwise procedures (which combined forward and backward selection techniques). The log-likelihood ratio (LR) test was used to confirm the variable selection. Excluded independent variables from the automatic methods were then confirmed manually. By running the model with linear regression and obtaining the variance-inflation-factors, all selected variables were evaluated for multicollinearity (VIF). A VIF value of 10 or greater was considered to have multicollinearity issues, which can distort the relationship between the independent variables, resulting in incorrect interpretation^30^.

Possible two-way interactions between significant independent variables were checked using interaction terms, which were created by the multiplication of two variables. Each term was added to the model one at a time and its significance was determined. The Hosmer-Lemeshow goodness-of-fit test (*p*-value of 1 indicates a perfect fit) was used to assess the fitness of the main effect model, while Cook’s Distance statistics (cutoff point = 1.0) was used to assess the presence of influential outliers. Data analyses were conducted using R (ver. 4.0.2) statistical software^31^.

Descriptive statistics, logistic regression, and Cook’s Distance analyses were performed with functions from the R “stats” package, while the stepwise variable selection was performed with the “stepAIC()” function from the “MASS” package. The LR and Hosmer-Lemeshow goodness-of-fit tests were run with functions from the “rms” and “ResourceSelection” packages, respectively. All tests were two-tailed, and statistical significance was defined as a *p*-value of less than 0.05.

## RESULTS

A total of 338 of the 374 questionnaires distributed were returned, representing a response rate of 90.4%. Table 2 summarizes the study participants’ characteristics by gender, year of study, and clinical level. The proportion of females was higher (68.3%), and more than half of the respondents (57.7%) were at the clinical level.

**Table 2:** Sample distribution by gender, year of study and clinical level (n=338).

Variables	n (%)	[95%CI]
**Gender**		
Female	231 (68.3)	[63.0, 73.2]
Male	107 (31.7)	[26.8, 37.0]
**Year of study**		
Year 1	76 (22.5)	[18.2, 27.4]
Year 2	67 (19.8)	[15.8, 24.6]
Year 3	64 (18.9)	[15.0, 23.6]
Year 4	76 (22.5)	[18.2, 27.4]
Year 5	55 (16.3)	[12.6, 20.7]
**Clinical level**		
Preclinical	143 (42.3)	[37.0. 47.8]
Clinical	195 (57.7)	[52.2, 63.0]

Note: CI = Confidence intervals.

The status of sleep quality and the distribution of various lifestyle levels are shown in Table 3, with 36.7% of subjects having PSQI scores greater than 5 (poor sleep quality).

**Table 3:** Sleep quality and health-related lifestyle behaviors of the study sample (n=338).

Variables	n (%)	[95%CI]
**Sleep quality**		
Good (PSQI≤5)	214 (63.3)	[57.9, 68.4]
Poor (PSQI>5)	124 (36.7)	[31.6, 42.1]
**Lifestyle domains**		
Health and exercise		
Healthy	73 (21.6)	[17.4, 26.4]
Moderate	158 (46.7)	[41.3, 52.2]
Unhealthy	107 (31.7)	[26.8, 37.0]
Psychological health		
Healthy	74 (21.9)	[17.7, 26.8]
Moderate	132(39.1)	[33.9, 44.5]
Unhealthy	132 (39.1)	[33.9, 44.5]
Substance use		
Healthy	221 (65.4)	[60.0, 70.4]
Moderate	75 (22.2)	[18.0, 27.1]
Unhealthy	42 (12.4)	[9.2, 16.5]
Nutrition		
Healthy	114 (33.7)	[28.8, 39.1]
Moderate	159 (47.0)	[41.6, 52.5]
Unhealthy	65 (19.2)	[15.2, 23.9]
Social concern		
Healthy	61 (18.0)	[14.2, 22.6]
Moderate	125 (37.0)	[31.9, 42.4]
Unhealthy	152 (45.0)	[39.6, 50.4]
Sense of purpose		
Healthy	95 (28.1)	[23.4, 33.3]
Moderate	118 (34.9)	[29.9, 40.3]
Unhealthy	125 (37.0)	[31.9, 42.4]

Notes: PSQI = Pittsburg sleep quality index; CI = Confidence intervals.

In terms of physical health and exercise, 158 subjects (46.7%) were categorized as moderate, while 107 subjects (31.7%) were categorized as unhealthy. For psychological health, a similar number of subjects [n=132 (39.1%)] were classified as moderate and unhealthy. When asked about substance use, more than half of the subjects [n=221 (65.4%)] reported leading a healthy lifestyle in terms of substance use, which included abstaining from harmful substances such as alcohol and tobacco. This was followed by 22.2% (n=75) of those classified in the moderate category. In terms of nutritional factors, 33.7% (n=114) of respondents reported practicing healthy habits, while 47.0% (n=159) and 19.2% (n=65) fell into the moderate and unhealthy categories, respectively. Further, the descriptive analysis revealed that 152 (45.0%) and 125 (37.0%) of the subjects, respectively, had unhealthy and moderate lifestyles in the social concern domain. On sense of purpose, 125 subjects (37.0%) were classified as unhealthy, while 118 subjects (34.9%) were classified as moderate.

The findings for the relationships between gender, clinical levels, and lifestyle factors, and sleep quality are shown in Table 4.

**Table 4:** Factors associated with sleep quality using logistic regression among the study sample (n=338).

	ULogR^a^		MLogR^b^		
**Variables**	**Crude** *OR* **(95% CI)**	*P* **value**	**Adj.** *OR^c^* **(95% CI)**	χ*^2^* **stat ^d^** *(df)*	*P* **value**
**Gender** Female Male	Reference 1.76 (1.10, 2.81)	0.019	Reference 1.72 (1.05, 2.83)^f^	4.61 (1)	0.032^f^
**Clinical level** Preclinical Clinical	Reference 1.20 (0.76, 1.88)	0.429	1.12 (0.68, 1.82)	- - -	0.662
**Health and exercise**					
Healthy	Reference		Reference	-	
Moderate	1.36 (0.24, 2.48)	0.318	1.12 (0.58, 2.22)	-	0.738
Unhealthy	1.94 (1.03, 3.66)	0.041	1.20 (0.58, 2.51)	-	0.630
**Psychological**					
Healthy	Reference		Reference	8.99 (2)	
Moderate	1.88 (0.97, 3.63)	0.062	1.60 (0.81, 3.15)^f^	1.86 (1)^e^	0.172^f^
Unhealthy	3.31 (1.73, 6.34)	<0.001	2.64 (1.34, 5.21)^f^	7.87 (1)^e^	0.005^f^
**Substance use**					
Healthy	Reference		Reference	-	
Moderate	1.12 (0.65, 1.93)	0.691	0.93 (0.51, 1.67)	-	0.802
Unhealthy	2.40 (1.23, 4.69)	0.010	1.68 (0.79, 3.60)	-	0.177
**Nutrition lifestyle**					
Healthy	Reference		Reference	8.24 (2)	
Moderate	1.37 (0.82, 2.30)	0.232	1.15 (0.67, 1.98)^f^	0.25 (1)^e^	0.614^f^
Unhealthy	2.69 (1.43, 5.07)	0.002	2.48 (1.28, 4.82)^f^	7.24 (1)^e^	0.007^f^
**Social concern**					
Healthy	Reference		Reference	-	
Moderate	0.67 (0.35, 1.28)	0.222	0.58 (0.28, 1.21)	-	0.147
Unhealthy	1.23 (0.67, 2.27)	0.498	0.78 (0.37, 1.64)	-	0.506
**Sense of purpose**					
Healthy	Reference		Reference	-	
Moderate	1.23 (0.68, 2.20)	0.491	1.00 (0.51, 1.98)	-	0.989
Unhealthy	1.94 (1.10, 3.42)	0.021	1.06 (0.52, 2.17)	-	0.871

Notes: ^a^Univariable logistic regression;

b Multiple logistic regression (the model fits reasonably well; model assumptions are met; no significant interaction terms, no multicollinearity problems, no influential outliers);

c Adjusted odds ratio;

d Likelihood ratio (LR) test;

e Wald test;

f Values from the main effect model.

Poor sleep quality was found to be significantly associated with gender (*p*=0.019), and unhealthy lifestyles in five components: health and exercise (*p*=0.041), psychosocial health (*p*<0.001), substance use (*p*=0.010), nutrition (*p*=0.002), and sense of purpose (*p*=0.021) in univariable analyses. At the multivariable level, gender (*p*=0.032) and factors related to psychological health (*p*=0.005) and nutrition (*p*=0.007) remained significant, whereas health and exercise, substance use, and sense of purpose were no longer significant. Males were 1.72 times (95%CI: 1.05, 2.83) more likely than females to have poor sleep quality, according to multiple logistic regressions. Subjects with unhealthy lifestyle behaviors related to psychological health and nutrition had 2.64 (1.34, 5.21) and 2.48 (1.28, 4.82) times higher odds of poorer sleep quality, respectively, when compared to those with healthy lifestyles.

## DISCUSSION

The purpose of this study was to investigate the relationship between lifestyle factors and sleep quality in undergraduate dental students at a Malaysian private university. We also analyzed the influence of gender and clinical stage on the outcome variable. The factors associated with sleep quality were identified using stepwise logistic regression.

In our study, sleep quality was assessed using the PSQI, with cutoff scores of >5 and £5 indicating poor and good sleep quality, respectively. The Cronbach’s alpha value of the PSQI scale in this study was low (0.56), which could be attributed to the homogeneity of the study subjects in terms of course enrollment, campus living environment, and ethnicity. According to Pike and Hudson (1998)^32^, homogeneous subject responses to a scale item can reduce the alpha value, and thus the instrument’s reliability should be interpreted cautiously when subjects have nearly similar experiences. It is worth noting that our alpha value is comparable to the values of 0.54, 0.56, and 0.59 reported in previous studies recruiting similarly healthy subjects^33-35^, and is thus acceptable for screening purposes.

The subjective self-reported proportion of students with poor sleep quality (PSQI>5) was 36.7%. Accordingly, one study found that 31.0% of a sample of Chinese college students had poor sleep quality^36^, while another study found that 33.8% of college students had the same problem^37^. Our prevalence, however, is considerably lower than that reported among dental students in Pakistan^38^, Brazil^39^, and Saudi Arabia^3^, which are 58.1%, 60.1%, and 72.5%, respectively. The wide variation could be attributed to cultural differences in sleeping habits as well as different coping strategies for sleep disruptions. Moreover, the duration, curriculum structure (e.g., clinical component and case requirements), and mode of delivery of dental education vary by institution, resulting in varying levels of burden and stress for students. Additionally, the learning environment is increasingly guided by self-directed learning to develop students’ capacity for lifelong learning, which can be challenging for those accustomed to didactic teaching^40^.

Poor sleep quality was found to be significantly related to gender in our study. Our study demonstrates that male subjects were at a greater risk of experiencing poor sleep quality than female subjects. Other studies using the PSQI in college or university samples, on the other hand, have found that female subjects have poorer sleep quality than their male counterparts^41-43^. Sleep is known to be influenced by a variety of factors, including stress, anxiety, and hormone-related factors, all of which are more prevalent in women^44,45^. Almost all students at this university live on campus in hostels with four students per room. Based on the authors’ personal communication with a subset of the sample, many of the male students stayed up late at night playing mobile phone games or checking social media, which could lead to a later bedtime and shorter sleep duration^46^. As a result, their roommates who wish to sleep early may find it difficult due to the noise from their late sleepers’ friends. However, in our study, this was not a major issue among female students.

It is commonly assumed that dental students in their clinical years will be subjected to a demanding schedule with a massive amount of material to learn. Furthermore, they are responsible for the patients in their care and are expected to demonstrate exceptional cognitive performance and manual dexterity^3^. Nonetheless, our study found that students in the clinical years had no significantly higher odds of poor sleep quality than their juniors in the preclinical years. One possible explanation is that both the preclinical and clinical phases present unique challenges, with comparable burdens at both levels.

The LHQ-B scale was used to assess lifestyle factors, with scores categorized as healthy, moderate, or unhealthy. This study identified two important domains associated with poor sleep quality: psychological health and nutrition. Notably, we found no evidence of significant associations between lifestyle components related to health and exercise, substance use, social concern, and sense of purpose and sleep quality at the multivariable level. This lack of significance is surprising, given that other studies have linked certain unhealthy behaviors, such as inactivity, substance abuse, a negative social outlook, and a lack of purpose, to poor sleep quality in a population of college students^47-49^.

The LHQ-B scale assesses the ability to cope with stress as one of the psychological health components. Previous studies have repeatedly demonstrated that dental students suffer from depression, stress, and anxiety^18,50,51^. Our findings indicated that students who exhibited an unhealthy psychological health pattern were 2.64 times more likely to have poor sleep quality than those with a positive psychological perspective. The dental program has extensive curricula and requires students to meet stringent academic and clinical requirements. Before taking the exam, students must complete the requirement for practical and clinical cases. This can cause a great deal of stress for the students, especially if they are behind schedule. Additionally, the cost of studying dentistry, particularly at a private university, is high, and the job market after graduation is uncertain^52^. All of these factors can lead to psychological distress, which can interfere with sleep.

Students with a poor nutrition-related lifestyle were nearly 2.5 times more likely to have poor sleep quality than those with healthy nutrition habits, according to our findings. This finding is consistent with a review of the literature that discovered reasonable evidence that healthy eating habits could improve sleep quality^53^. According to a previous study on a sample of Malaysian university students, nearly 60% of them consumed a high-fat diet at least four times a week, and roughly three-quarters of the sample ate 1-2 servings of fruits or none at all each day^54^. Living in student hostels may have exaggerated the role of nutrition on sleep quality in our sample. The limited and repetitive menu of meals available at campus eateries may explain this. The majority of the meals were Indian and Malay, with some being high in carbohydrates and fats. A high-carbohydrate diet, in particular, can have a negative impact on sleep quality^55^. To compound matters, because the university is located quite far away from the city, access to nutritious food with a variety of options may be even more limited. Notably, diet and sleep quality have been proposed to have a two-way relationship, meaning that food can influence sleep quality while sleep can also influence nutritional intake via biological and behavioral mechanisms^56^.

We assume that the interplay of gender and certain lifestyle factors can influence sleep quality from a clinical point of view. A systematic review found a link between gender, stress, sleep, and the risk of obesity in young people^57^. It was discovered that the direction of body mass index change for each gender can differ depending on the type of life event stressor, and that concurrent sleep disruptions exacerbate the impact of stressors on metabolic outcomes. Grzymisławska et al. (2020)^58^ reported that gender-specific nutritional preferences exist, and they are influenced by a variety of factors, including psychological ones. Accordingly, we checked for potential two-way interactions on sleep quality between the significant independent variables in our study (gender, psychological health, and nutrition lifestyle). However, none of the interaction terms were significant.

There are some limitations to this study. The cross-sectional design of the study precludes inferring causal relationships. Furthermore, using self-reported questionnaires to assess sleep quality and lifestyle factors may lead to reporting bias. We also did not collect age information, which could potentially confound the associations discovered in this study. According to research, older people have poorer sleep quality, which is primarily attributed to aging factors such as health issues, polypharmacy with or without medication side effects, as well as psychosocial issues^59^. Nonetheless, given the relatively young individuals and the narrow age difference of our subjects (the dental students at our university are typically between the ages of 19 and 24 years), we believe this confounding effect is minimal, as confirmed by Angelin et al. (2020)^60^.

## CONCLUSION

In our study, the prevalence of poor sleep quality among undergraduate dental students is comparable to levels reported in other studies. Male students were more likely than female students to have poor sleep quality. The hypothesis that certain lifestyle factors have a significant relationship with sleep quality was confirmed. Our findings indicated that poor sleep quality (PSQI score >5) was associated with unhealthy lifestyle habits across all lifestyle domains, but the association was statistically significant for psychological health and nutrition. As a result, health education focusing on these factors could be offered to dental students with poor sleep quality as a strategy for improving their lifestyle habits and sleep quality.
